# Accuracy of Handheld Blood Glucose Meters at High Altitude

**DOI:** 10.1371/journal.pone.0015485

**Published:** 2010-11-12

**Authors:** Pieter de Mol, Hans G. Krabbe, Suzanna T. de Vries, Marion J. Fokkert, Bert D. Dikkeschei, Rienk Rienks, Karin M. Bilo, Henk J. G. Bilo

**Affiliations:** 1 Department of Internal Medicine, Canisius Wilhelmina Hospital, Nijmegen, The Netherlands; 2 Department of Clinical Chemistry, Isala Clinics, Zwolle, The Netherlands; 3 Department of Cardiology, Isala Clinics, Zwolle, The Netherlands; 4 Centre for Human Aviation, Dutch Airforce, Soesterberg, The Netherlands; 5 Department of Internal Medicine, Isala Clinics, Zwolle, The Netherlands; 6 Department of Internal Medicine, University Medical Centre, Groningen, The Netherlands; Cardiff University, United Kingdom

## Abstract

**Background:**

Due to increasing numbers of people with diabetes taking part in extreme sports (e.g., high-altitude trekking), reliable handheld blood glucose meters (BGMs) are necessary. Accurate blood glucose measurement under extreme conditions is paramount for safe recreation at altitude. Prior studies reported bias in blood glucose measurements using different BGMs at high altitude. We hypothesized that glucose-oxidase based BGMs are more influenced by the lower atmospheric oxygen pressure at altitude than glucose dehydrogenase based BGMs.

**Methodology/Principal Findings:**

Glucose measurements at simulated altitude of nine BGMs (six glucose dehydrogenase and three glucose oxidase BGMs) were compared to glucose measurement on a similar BGM at sea level and to a laboratory glucose reference method. Venous blood samples of four different glucose levels were used. Moreover, two glucose oxidase and two glucose dehydrogenase based BGMs were evaluated at different altitudes on Mount Kilimanjaro. Accuracy criteria were set at a bias <15% from reference glucose (when >6.5 mmol/L) and <1 mmol/L from reference glucose (when <6.5 mmol/L). No significant difference was observed between measurements at simulated altitude and sea level for either glucose oxidase based BGMs or glucose dehydrogenase based BGMs as a group phenomenon. Two GDH based BGMs did not meet set performance criteria. Most BGMs are generally overestimating true glucose concentration at high altitude.

**Conclusion:**

At simulated high altitude all tested BGMs, including glucose oxidase based BGMs, did not show influence of low atmospheric oxygen pressure. All BGMs, except for two GDH based BGMs, performed within predefined criteria. At true high altitude one GDH based BGM had best precision and accuracy.

## Introduction

Regular exercise and a healthy life style should be part of daily life of everybody, but definitely of any person with diabetes mellitus. Minimal requirements for exercise have been formulated, with a minimum of 30 minutes of vigorous exercise or brisk walking, five times weekly. However, increasing numbers of people with diabetes do participate in more strenuous forms of physical activity, amongst others high-altitude trekking and mountain climbing. These kinds of activities do pose special challenges to subjects with diabetes mellitus treated with insulin. Glucose levels have to be kept under good control, and it is a challenge to find a balance between energy intake, energy expenditure, blood glucose levels, and insulin requirements. In general, frequent assessment of blood glucose levels will be necessary to allow proper adjustments.

Therefore, both accuracy and easy accessibility of frequent blood glucose measurements in subjects with type 1 diabetes mellitus are paramount for a safe activity at high altitude.

Previous studies have examined the accuracy and reliability of various glucose meters at (simulated) altitudes up to 5,000 m. These and other studies with type 1 diabetes mellitus patients indicated a considerable subset of the handheld blood glucose meters (BGMs) to be unreliable at high altitude [1]–[6]. One could hypothesize that glucose oxidase (GOX) BGMs, which use oxygen as one of the substrates, would underestimate true glucose values since less oxygen is available under hypobaric conditions. This effect has been demonstrated by some [5]. Others have demonstrated oxygen independent BGMs using glucose dehydrogenase (GDH), to perform better under hypobaric conditions and GOX based BGMs to underestimate true blood glucose levels [1]. However, these studies were limited by field- or simulated altitude settings only [3]–[5], test protocols using only GOX based BGMs [3], test protocols up to simulated altitudes <4500 m [3]–[5] or with outdated material [3], [4].

Since we planned an expedition to climb Mount Kilimanjaro with a team of people with type 1 diabetes mellitus, we were in need of the most reliable handheld BGMs up to 6000 m altitude. The purpose of this study was to investigate whether modern GDH or GOX based BGMs are reliable under simulated and true high altitude conditions.

We hypothesized, that GDH based BGMs would be more accurate than GOX based BGMs at high altitude, since GDH based BGMs are by definition less oxygen dependent in their reaction than GOX based BGMs.

## Methods

Two experiments were performed; First, all BGMs were tested at simulated altitudes in a hypobaric chamber. Second, a selection of GOX and GDH based BGMs in the first experiment were used during the ascent of Mount Kilimanjaro (5895 m).

The local ethics committee of the Isala Clinics Zwolle, the Netherlands, approved the study protocol and all participants gave written informed consent.

### Hypobaric chamber experiment

We used a hypobaric chamber of the Dutch Airforce Research facility in Soesterberg, The Netherlands. Six GDH and three GOX based BGMs were tested at simulated altitudes: sea level, 2000 m, 3000 m, 4000 m and 5000 m while temperature (20°C) and humidity were kept constant. GDH based BGMs used were; Freestyle Mini and Precision X-ceed (both Abbott Diabetes Care, USA), Hemocue 201+ (Quest Diagnostics U.K.), Accu-Chek Aviva and Accu-Chek Compact Plus (both Roche Diagnostics, Switzerland) and the Contour Link (Bayer, Germany). GOX based BGMs used were; Glucocard Memory (Menarini Diagnostics, Italy) Statstrip (Nova Biomedical, USA) and the Klinion (Klinion Diabetes Care, Medeco the Netherlands) All BGMs used were new - i.e. the same BGM was not extensively used for medical practice before the experiments took place - to prevent errors from wear and tear. Venous blood drawn from a healthy individual spiked to a glucose concentration of 5, 10, 15 and 20 mmol/L was used as substrate to test BGMs. True glucose values were determined with the laboratory certified Glucose Hexokinase (GHex) based method (Roche Diagnostics, Manheim, Germany). This GHex method is aligned with the GC-MS glucose reference method. BGMs were simultaneously tested inside and outside the hypobaric chamber, and samples were tested in duplicate.

To prevent glucose variation due to time-dependent glycolysis by cellular uptake in blood samples (5–7% per hour [7]) a blood sample was prepared for GHex reference testing using a perchloric acid preparation method. The latter was done at the time spiked glucose samples were prepared – prior to BGM and GHex testing. These blood samples were used for both GHex and BGM glucose testing under normobaric and hypobaric conditions.

### Field experiment

On Mount Kilimanjaro testing took place at incremental altitudes (range 1300–4600 m), using capillary blood samples from eight subjects with type 1 diabetes and eight healthy control subjects. Care was taken to take a full droplet of blood from a subject's warm and clean index finger. Four BGMs, two GDH based BGMs (Accu-Chek Compact Plus and Contour) and two GOX based BGMs (Klinion and Glucocard) were used. Samples were tested at temperatures ranging from ±10–30°C using one single sample of each subject. GHex reference testing was not available at altitude. Instead, standard reference solutions (glucose range 2.4–4.1; 4.4–6.7 and 14.0–17.9 mmol/L; NOVA biomedical, USA) were used to test within-BGM performance at different altitudes. These control solutions are made for the Statstrip BGM and were primarily used to observe possible consistent biases within that same BGM (precision) with repeated measures at different altitudes. For between-BGM performance comparison the best-tested BGM from the hypobaric chamber experiment –the GDH based Accu-Chek Compact Plus- was used as a reference. No air humidity was measured at altitude.

### Analysis and statistics

Performance criteria for BGMs were set at a difference of ±1 mmol/L of the reference method when <6.5 mmol/L, or ±15% when the reference sample read ≥6.5 mmol/L (Dutch Organisation of Applied Physics and Science (“TNO”) guideline, the Netherlands). Furthermore, Clarke's error grid analysis was used to determine clinically relevant accuracy. [8] The error grid plots the reference glucose and the glucose measured by the BGM on a x-y plot. The grid is divided into 5 zones (A–E) of clinical accuracy and corresponding treatment assumptions to correct the glucose value. Zone A represents the target glucose range and glucose values are clinically accurate; Zone B represents glucose values that deviate >15% from the reference glucose leading to no or benign treatment; Zone C glucose values would lead to overcorrection of acceptable glucose values causing hyper- or hypoglycemia; Zone D represents glucose values that could lead to dangerous misdetection and failure to treat; Zone E glucose values are opposite from the reference glucose leading to very dangerous treatment decisions. Values in zone A and B are considered clinically acceptable. Glucose measured by BGMs is presented in means (mmol/L) ± SEM unless stated otherwise. Deviation of mean BGM glucose from the reference method is presented in percentage (%) and it's range. Overestimation is presented as the actual percentage and underestimation is preceded by a minus (-) sign.

## Results

### Hypobaric chamber experiment

Each sample was tested in duplicate under hypobaric and normobaric conditions simultaneously, and results are presented as the mean glucose levels with absolute (mmol/L) or relative (%) bias from GHex glucose determination.

Four of six tested GDH and all three GOX based BGMs performed within predefined performance criteria of ±1.0 mmol/L when reference glucose was <6.5 mmol/L and ±15% when reference glucose was >6.5 mmol/L at all simulated altitudes. There were no mechanical failures due to hypobaric conditions.

One GDH based BGM (Freestyle mini) showed biases (range 15.2 to 18.5%) outside of predefined criteria for 7 out of 20 tested glucose samples at simulated altitudes of 2000 m and 3000 m. Also, this BGM showed biases outside of predefined criteria at 4 out of 20 sea level readings (range 16.0 to 18.5%).

**Figure 1:**
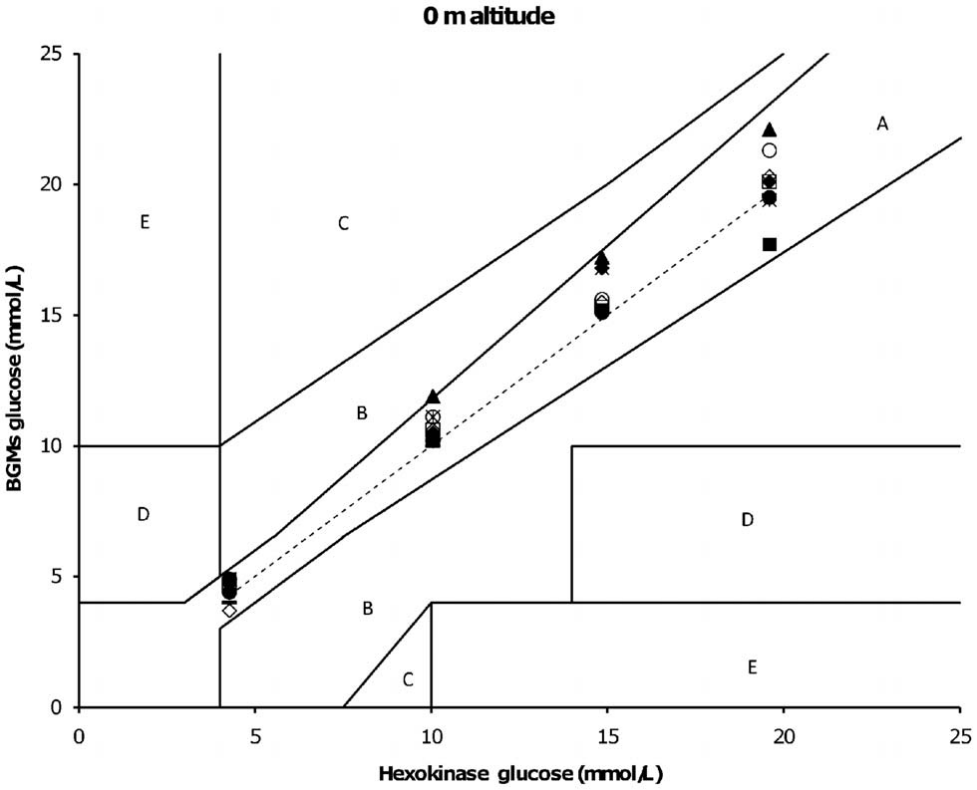
Error grid analysis of 5, 10, 15 and 20 mmol/L glucose testing samples of BGMs (y- axis) against reference (GHex) (x-axis) at sea level. Note: Leftward deviation from dashed line means overestimation of true glucose by tested BGM compared to GHex reference glucose and vice versa. No differences were noted outside of predefined criteria due to testing conditions at sealevel (0 m) in and outside the hypobaric chamber. Black diamonds  =  Contour; Black squares  =  Accu Chek Aviva; Black circles  =  Accuchek Compact Plus; Black triangles  =  Freestyle Mini; Black asterixes  =  Precision; Black stripes  =  Hemocue; White squares  =  Klinion; White circles  =  Statstrip; White diamonds  =  Glucocard; Dashed line  =  GHex reference glucose.

**Figure 2:**
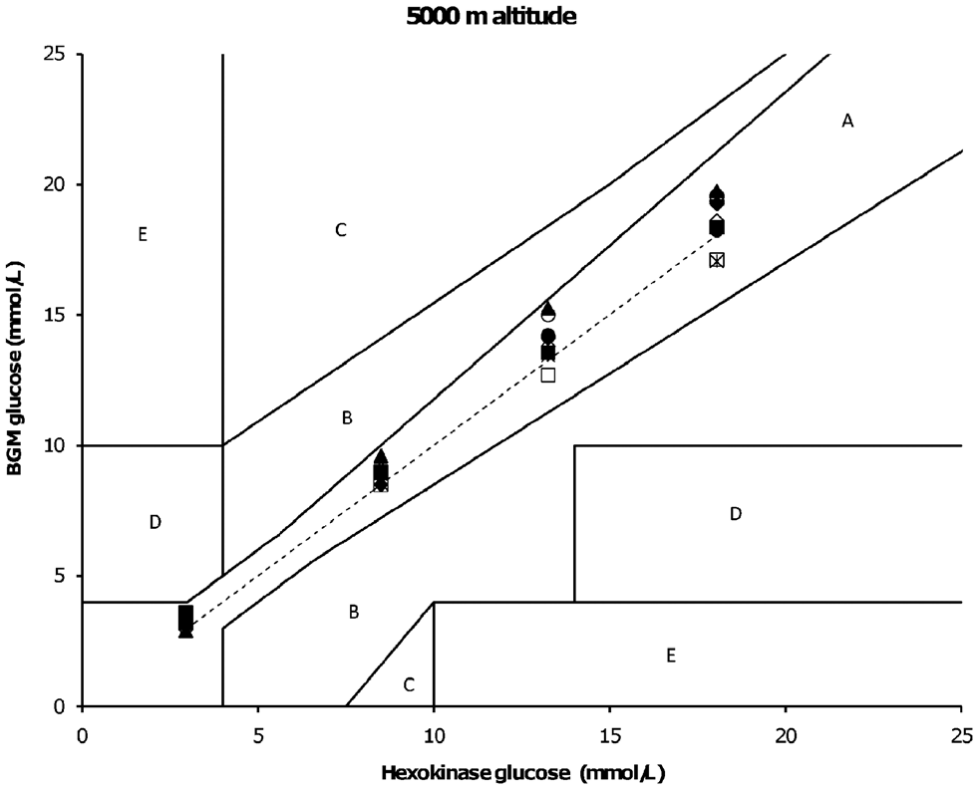
Error grid analysis of 5, 10, 15 and 20 mmol/L glucose testing samples of BGMs (y- axis) against reference (GHex) (x-axis) at 5000 m simulated altitude. Note: Leftward deviation from dashed line means overestimation of true glucose by tested BGM compared to GHex reference glucose and vice versa. Black diamonds  =  Contour; Black squares  =  Accu Chek Aviva; Black circles  =  Accuchek Compact Plus; Black triangles  =  Freestyle Mini; Black asterixes  =  Precision; Black stripes  =  Hemocue; White squares  =  Klinion; White circles  =  Statstrip; White diamonds  =  Glucocard; Dashed line  =  GHex reference glucose.

Furthermore, another GDH based BGM (Precision X-ceed) only showed biases outside of predefined criteria at sea level for 5, 10 and 15 mmol/L glucose samples (Table 1). In general, under hypobaric conditions (at simulated altitude) both the GOX and GDH based BGMs tended to overestimate glucose levels compared to normobaric conditions (bias range; GDH BGMs: −8.2 to 16.9% and GOX BGMs: 0 to 10.8%) (Figure 1, 2 and Table 1, 2). Table 1 and 2 demonstrate the relative bias (%) of the 10 mmol/L test sample, and is representative for the remaining tested glucose samples (5, 15 and 20 mmol/L).

**Table 1:** Relative bias (%) of GDH based BGMs compared to the Hexokinase laboratory reference method at different simulated altitudes (10 mmol/L glucose sample).

GDH	Hexokinase *	Contour Link		Accu-chek Aviva		Accu-chek Compact Plus		Freestyle Mini		Precision Xceed		Hemocue	
Altitude (m)	Glucose (mmol/L)	normo (%)	hypo (%)	normo (%)	hypo (%)	no rmo (%)	hypo (%)	normo (%)	hypo (%)	normo (%)	hypo (%)	normo (%)	hypo (%)
0	10.05	3.8	4.3	3.8	1.5	2.9	1.5	2.4	15.5	11.1	9.5	−2.6	−0.5
2000	9.73	−4.0	−3.5	2.3	3.7	2.8	0.8	10.4	16.9	15.4	6.5	−2.9	−0.8
3000	9.38	−2.5	2.8	0.8	−3.0	2.8	2.8	12.8	15.2	15.9	8.1	−6.5	−3.0
4000	8.93	3.0	4.5	−0.3	6.1	3.0	3.5	18.5	8.9	12.9	−8.2	−0.3	3.5
5000	8.50	6.1	0.0	6.1	5.0	4.5	4.5	17.1	11.5	12.8	0.6	2.9	7.6

Note: normobaric BGMs stay at sea level. (normo =  bias under normobaric conditions (sea level); hypo =  bias under hypobaric conditions (simulated altitude),

* =  reference method).

**Table 2:** Relative bias (%) of GOX based BGMs compared to the Hexokinase laboratory reference method at different simulated altitudes (10 mmol/L glucose sample).

GOX	Hexokinase *	Statstrip		Klinion		Glucocard	
Altitude(m)	Glucos(mmol/L)	normo(%)	hypo(%)	normo(%)	hypo(%)	normo(%)	hypo(%)
0	10.05	12.6	9.5	−5.8	5.2	2.9	5.2
2000	9.73	14.7	10.8	−7.5	0.8	3.7	3.7
3000	9.38	11.1	5.8	−2.5	3.4	−5.3	4.8
4000	8.93	4.5	6.1	−0.8	0.8	3.5	6.1
5000	8.50	2.3	5.6	−2.4	0.0	4.5	6.6

Note: normobaric BGMs stay at sea level. (normo =  bias under normobaric conditions (sea level); hypo =  bias under hypobaric conditions (simulated altitude),

* =  reference method).

As a group phenomenon, no significant difference was observed between GOX- and GDH-BGMs under hypobaric conditions (Bias at 2000–5000 m: 5.8±7.9 (−5.0 to 19.1) vs. 7.5±6.5 (−3.5 to 22.1) respectively (mean % ± SEM (range %) (p = NS)).

Indeed, differences observed were seen in all tested glucose concentrations and were therefore not sample dependent.

Moreover, no trend in bias was seen for the individual BGMs with increasing simulated altitude. However, one GDH-based BGM performed better under hypobaric than under normobaric conditions (Precision Xceed).

The best performing BGM under hypo- and normobaric conditions and at all glucose concentrations tested was the GDH based BGM Accu-chek Compact Plus. When further assessing the specific results of the Accu-chek Compact Plus, there was a trend of greater overestimation of 10 and 15 mmol/L glucose samples at higher simulated altitudes. Furthermore, the smallest of biases was found in the 20 mmol/L sample. However, the bias of this BGM (bias range −1.1 to 6.7% at all glucose concentrations and simulated altitudes) was well within predefined criteria for acceptable bias and within meter variation was minimal. Moreover, the meter was user-friendly, which is important, especially under extreme conditions such as high altitude mountaineering.

### Field experiment

Three standard glucose control solutions were used as a crude reference method of BGM performance at Mt Kilimanjaro at altitudes of 1300 m, 3000 m, 3700 m and 4600 m. Also one single sample of capillary blood of eight subjects with type 1 diabetes mellitus and eight healthy control subjects was used to test BGMs at altitudes of 1300 m, 3000 m, 3770 m, 3900 m and 4600 m altitude.

Measurements with the standard glucose control solutions demonstrated consistent results of all BGMs except for the Klinion GOX based BGM which showed a large within BGM variance at different altitudes in all glucose ranges tested (Table 3). However, when analyzing BGM accuracy in relation to the glucose solutions reference measurements one GOX based BGM (Glucocard) consistently underestimated true glucose measurement especially in the lower glucose range (2.4 to 4.4 mmol/L). These results were in accordance with results previously obtained in the hypobaric chamber.

**Table 3:** GOX and GDH based BGMs at Mount Kilimanjaro at different altitudes tested with standard glucose solutions.

Altitude	Control solution	GDH BGMs		GOX BGMs	
(m)	Glucose (mmol/L)	Glucose (mmol/L)		Glucose (mmol/L)	
		Accu-chek Compact Plus	Contour	Klinion	Glucocard
1300	2.4–4.1	3.9	2.7	4.6	1.9
	4.4–6.7	5.6	5.5	6.0	4.7
	14.0–17.9	14.7	15.7	17.5	14.7
3000	2.4–4.1	3.9	3.0	3.0	1.6
	4.4–6.7	5.9	5.6	5.6	4.3
	14.0–17.9	14.9	16.6	15.5	14.2
3700	2.4–4.1	3.9	2.5	3.2	1.8
	4.4–6.7	5.4	5.7	5.9	4.4
	14.0–17.9	14.5	15.5	16.4	13.8
4600	2.4–4.1	3.8	3.1	2.6	1.4
	4.4–6.7	6.0	5.9	4.9	4.2
	14.0–17.9	14.9	16.2	13.9	14.0

**Figure 3:**
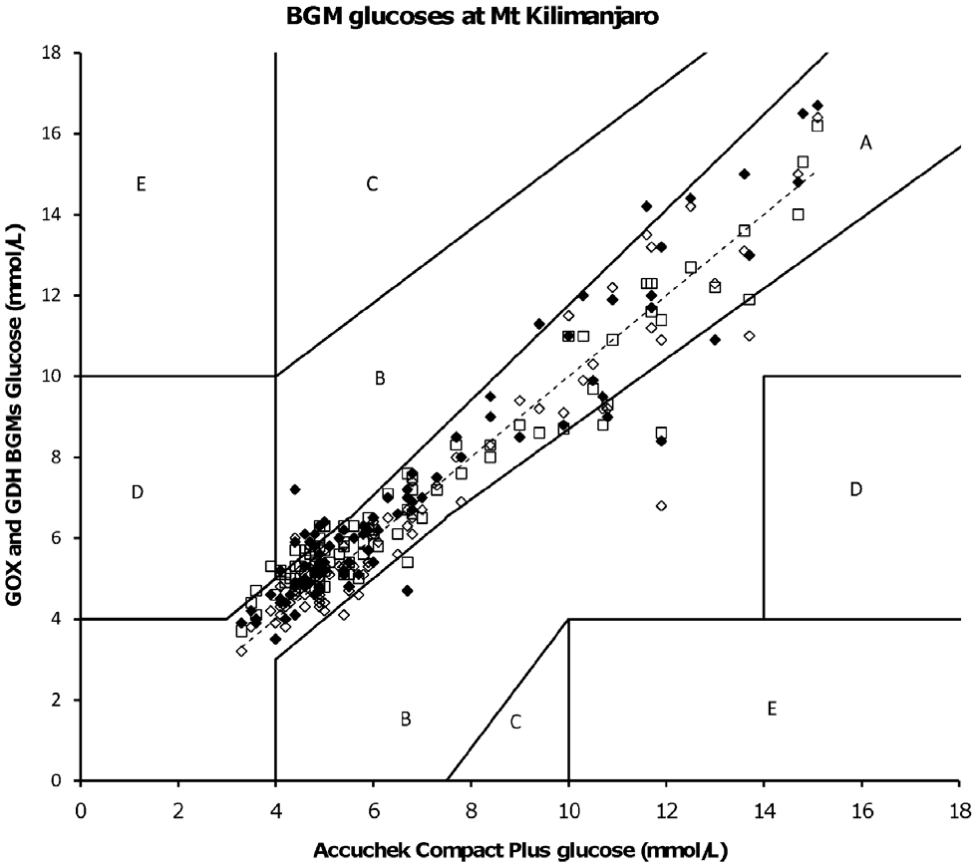
Error grid analysis of glucose samples of all (healthy controls and diabetes type 1) subjects measured by GOX- and GDH-BGMs (y-axis) at different altitudes on Mount Kilimanjaro (range 1300–4600 m) compared to Accuchek BGM glucose as a reference method (x-axis); (mmol/L). Note: leftward deviation from dashed line indicates overestimation of glucose measured by BGM and rightward deviation means underestimation by BGM compared to Accu-Chek BGM. Black diamonds  =  Contour; White squares  =  Klinion; White diamonds  =  Glucocard; Dashed line  =  Accu Chek Compact Plus BGM as a reference glucose.

When comparing BGMs on Mount Kilimanjaro with the best tested BGM in the hypobaric chamber (Accuchek Compact Plus; a GDH based BGM) for capillary glucose samples of subjects with type 1 diabetes mellitus and healthy controls at altitudes ranging 1300–4600 m, a total of 228 paired glucose measurements were analyzed. Of these, 47 (21%) were outside of set performance criteria primarily observed in the 3.6 to 5.2 mmol/L mean glucose range, and specifically due to an overestimation (Fig 3). In comparison to the reference BGM, the Glucocard, a GOX based BGM, performed better than the Contour (GDH) and the Klinion (GOX) BGMs (3 vs. 10 vs. 8% of bias outside performance criteria respectively). Most BGM performance criteria violations were observed at altitudes of 3000–3900 m.

## Discussion

The present study was conducted to test accuracy and reliability of GOX and GDH based BGMs at simulated and true high altitude. We hypothesized that oxygen independent GDH based BGMs would be more reliable and accurate at high altitude.

In contrast to previous reports and our hypothesis, no significant differences were observed between GOX- and GDH-based BGMs at simulated high altitude in all tested glucose ranges.

At true high altitude within-meter variation and accuracy compared to standard reference glucose solutions was better in the GDH based BGMs, and best in the Accu-Chek Compact Plus BGM, independent from glucose solutions used.

In general, most BGMs tended to overestimate blood glucose at high altitude.

At simulated high altitude one GDH based BGM performed better under hypobaric conditions (Precision Xceed), possibly due to in-meter failure of the normobaric BGM. This is suggested by the fact that under normobaric conditions (sea level) this BGM showed constant biases close to 15% in the 5, 10 and 15 mmol/L glucose samples. Furthermore, the most inaccurate BGM in all glucose ranges was the GDH based Freestyle Mini (28% of total glucose readings > +15% bias) without an apparent effect of altitude.

Lastly, there was a time dependent glucose lowering effect seen in the reference samples (GHex) possibly due to cellular uptake despite perchloric acid preparation of samples. (Table 1, 2) As expected, a parallel decrement in glucose was observed when testing all BGMs.

At true high altitude, the GOX based Glucocard BGM had good within-meter variation and was reasonably accurate compared to the reference BGM. However, when testing with standard reference glucose solutions, it constantly showed underestimation of the lowest glucose reference sample and was at the low end of the other reference samples. This is not well explained by chemical technique used as the other GOX based BGM (Klinion) did not show this phenomenon. A possible explanation might be that substances in the reference glucose solution or errors in test strips interfered with glucose measurement in this meter. [9]

Overestimation of glucose by BGMs at true altitude could have dangerous consequences when hypoglycemia is falsely not shown by BGM readings and hypoglycemic symptoms might not be fully sensed due to extreme conditions. At the hyperglycemic range, the therapeutic decisions made due to overestimation of true blood glucose have less consequences than in the hypoglycemic range, where not treating hypoglycemia could lead to life threatening conditions. In this perspective, the findings of underestimation by the Glucocard GOX based BGM in the low glycemic range are not considered as dangerous as vice versa. On the contrary, if overestimation of glucose by BGMs at simulated altitude is truly present at true altitude this might lead to dangerous situations. However, at simulated altitude overestimation was limited to 17 (9%) of all measurements, all of which fell in the lower B zone of the error grid leading to no or benign treatment errors.

Research of BGMs at simulated high altitude up to 4000 m by Gautier et al. has reported underestimation up to −28.9% using venous blood samples of glucose ranges from 1.5 up to 26.3 mmol/L. However, this study did not differentiate between GOX or GDH based BGMs and BGMs used are outdated today. [4] Furthermore, Öberg and Östenson tested four GDH and one GOX based BGM at simulated and true high altitude up to 4500 and 5895 m respectively. Their study reported overestimation ranging from 6.5 up to 15% of the GOX based BGM at normo- and hyperglycemic glucose levels at 4500 and 2500 m. GDH based BGMs performed better and overestimated true glucose levels by 0.8 to 6.5% [1]. Interestingly, two similar GDH based BGMs were used and bias observed did not correspond with our study; the Contour (GDH based) BGM in their study showed an underestimation (−1.9 to −4.2%) and the Freestyle (GDH based) BGM only showed a bias of +0.8% of both ∼5.8 and ∼16.5 mmol/L glucose samples. When GDH based BGMs were tested at true high altitude a wide range of bias was reported and no reference method was used.

Our results differ from the findings in previous research. First, we did not find a significant method-related difference between GOX and GDH based BGMs at simulated high altitude. Second, although not compared to a laboratory reference method, we did not find a wide range of bias in GOX and GDH based BGMs up to 4600 m at true high altitude.

A possible explanation for the first difference observed in contrast to the study of Gautier et al. [4] might be improved oxygen-based technique and sensitivity of the reagent for oxygen. This is reflected by the fact that it takes less time to read out glucose test strips results now than it took thirteen years ago, suggesting that GOX based BGMs need less oxygen in order to show a proper reaction.

In our study, BGMs were tested at simulated high altitude under constant temperature and humidity, using a laboratory reference method. Previous research reported low temperatures and humidity influence accuracy of BGMs independent of altitude [1]–[2]. This might have compromised field research at true high altitude and explained the wide range of bias in cold conditions at 5895 m in the other studies. [1], [2]. Since at 4600 m we tested BGM's at 28°C, the difference in temperature might explain this contrasting result.

This study has limitations that might influence its results.

First, on Mount Kilimanjaro we could only test BGM's on within meter variation and relative bias compared to the best tested BGM, but not on true bias due to the lack of a laboratory reference method at high altitude. Also, the reference solution used was not compared to a laboratory reference method upon return from altitude. However, BGMs showed consistent findings that were in good accordance with results obtained with the hypobaric chamber experiment.

Second, on Mount Kilimanjaro blood glucose levels of tested BGMs were compared to the best performing BGM as a reference method based on results in a hypobaric chamber under constant environmental conditions. One could speculate that alternating temperatures and humidity on a mountain might influence the accuracy of tested BGMs and the accuracy of the reference BGM in particular, thereby compromising results. However, when testing with standard glucose solutions, the reference BGM showed best accuracy and within meter variation (Table 3). Moreover, in our study we tested BGMs at temperatures ranging from 10 to 28°C and these are well within the range of temperatures stated by BGM manufacturers to provide reliable glucose measurements.

Based on the tests in the hypobaric chamber and at true high altitude we concluded that the Accu-check Compact Plus GDH based BGM was most accurate at simulated altitude and most precise at true high altitude.

### Conclusion

No differences were observed between GDH and GOX based BGMs at simulated altitude up to 5000 m. All of the tested BGMs, except for two GDH based BGMs, performed within defined criteria for acceptable accuracy at simulated altitude. In general, at simulated high altitude BGMs tend to overestimate true glucose levels. At true high altitude GDH based BGMs performed better in relation to within-meter variation and accuracy.

If a similar effect of overestimation is present at true high altitude this could have dangerous consequences in the normo- and hypoglycemic range. Therefore, true high altitude studies with approved laboratory reference methods with measurement of humidity and temperature as possible confounders are warranted.
